# *Hepatozoon martis* in Italy: first evidence based on histopathological characterization and molecular confirmation in *Martes foina*

**DOI:** 10.1186/s13071-026-07384-3

**Published:** 2026-04-08

**Authors:** Filippo Maria Dini, Alicia Rojas, Luisa Vera Muscatello, Roberta Galuppi, Debora Torri, Mara Scremin, Patrizia Bassi, Giulia Maioli

**Affiliations:** 1 Department of Veterinary Medical Sciences, Via Tolara Di Sopra 50, Ozzano Dell’Emilia, Bologna, Italy; 2 Laboratory of Helminthology, Faculty of Microbiology, University of Costa Rica, San José, Costa Rica; 3 Centro de Investigación en Enfermedades Tropicales, University of Costa Rica, San José, Costa Rica; 4 Istituto Zooprofilattico Sperimentale Della Lombardia E Dell’Emilia-Romagna, Brescia, Italy

**Keywords:** *Hepatozoon martis*, Mustelidae, Tick-borne protozoa, Wildlife reservoirs, Phylogenetics

## Abstract

**Background:**

*Hepatozoon martis* is an apicomplexan parasite infecting mustelids, but its geographical distribution and pathological relevance remain incompletely understood. To date, its presence in Italy has not been documented. This study aimed to investigate the occurrence of *H. martis* in martens from northern and central Italy using an integrated pathological and molecular approach.

**Methods:**

Between 2023 and 2025, carcasses of 17 martens (*Martes* spp.) collected within wildlife health surveillance programs were examined. Tissue samples were screened for apicomplexan DNA by qPCR, and conventional PCR targeting the 18S rDNA, followed by sequencing and phylogenetic analyses, were applied for species characterization. Histopathological examinations were performed on tissues from freshly deceased animals.

**Results:**

Eight of 17 martens (47%), 16 identified as *Martes foina* and one *Martes martes*, tested positive for *Hepatozoon* infection. Sequencing confirmed *H. martis* in multiple tissues, including spleen, heart, lymph nodes, and muscle. Histopathological analysis revealed multifocal granulomatous myocarditis and myositis, with intracellular zoites and type II meronts observed within inflammatory lesions. Phylogenetic analyses of short and long 18S rDNA fragments showed that Italian isolates clustered with *H. martis* sequences from other European countries, displaying low genetic variability. *Hepatozoon* DNA was also detected in one *Ixodes* sp. collected from an infected animal.

**Conclusions:**

This study provides the first evidence to our knowledge of *H. martis* infection in Italy and highlights its pathogenic potential in stone martens. The low genetic diversity observed among European isolates suggests widespread circulation and gene flow across regions. Further investigations are required to elucidate transmission pathways, identify competent vectors, and assess the pathological significance of *H. martis* in wildlife.

**Graphical Abstract:**

**Figure:**
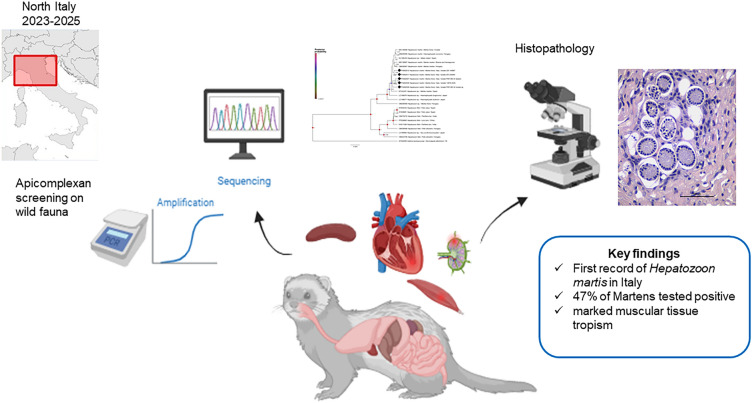

**Supplementary Information:**

The online version contains supplementary material available at 10.1186/s13071-026-07384-3.

## Background

Several *Hepatozoon* species are known to circulate among mammals in Europe, including *Hepatozoon canis*, *H. martis*, *H. felis*, *H. silvestris*, *H. sciuri*, *H. erhardovae*, and other novel genotypes (e.g. *Hepatozoon* sp. SK3, BV2) [1]. Among these, *H. canis* is the most widespread, infecting both domestic and wild canids (gray wolves, red foxes, and hunting dogs) with high prevalence in Southern and Northern Italy as well as Southwestern Europe [2–4]. Although often subclinical, *H. canis* can cause anemia, leucocytosis, and severe illness in dogs, particularly in the presence of comorbidities [2, 4]. Transmission occurs mainly via ingestion of infected ticks, primarily *Rhipicephalus sanguineus*, although other genera such as *Dermacentor* and *Ixodes* are also implicated [1].

*Hepatozoon felis* and *H. silvestris* are the principal species infecting felids. The latter has recently emerged in Italy and other European regions, producing both subclinical and clinical outcomes in domestic cats, including atypical presentations such as intestinal intussusception and myopathy [5–7]. The transmission pathways of these species remain uncertain, with both arthropod vectors and carnivory of paratenic hosts proposed as possible routes [8]. Despite the limited population size of European wildcats, this species may act as a reservoir for *Hepatozoon* infections. Coinfections with other protozoans, notably *Cytauxzoon europaeus*, have also been reported, underscoring the potential complexity of hemoparasite circulation in these hosts [8]. Rodent-associated *Hepatozoon* species, including *H. erhardovae* and several novel genotypes, circulate in small mammals, with fleas (e.g. *Ctenophthalmus* spp., *Megabothris turbidus*) serving as invertebrate hosts for some, while others may rely on alternative arthropod hosts or non-vectorial transmission pathways [9]. These dynamics highlight the ecological complexity of *Hepatozoon* transmission, in which wildlife reservoirs and diverse arthropod vectors support parasite persistence and facilitate potential spillover to domestic hosts. Indeed, cophylogenetic analyses have recently shown that host switching, rather than strict cospeciation, is the predominant driver of *Hepatozoon* evolution, underlining their capacity to exploit novel vertebrate hosts [10].

Within this framework*, H. martis* has been characterized as a distinct parasite of European mustelids. Multi-country surveys have documented infections predominantly in stone martens (*Martes foina*) and pine martens (*M. martes*) in Hungary [8], the Czech Republic [11], Bosnia and Herzegovina and Croatia [12], and Spain [13]. Infections have also been reported in other wild mustelid and non-mustelid species [13]. *Haemaphysalis concinna* has been proposed as a potential tick vector; however, its vector competence has not yet been experimentally confirmed [8, 12]. Histopathological examinations of previous reports reveal tissue cysts, myositis, and myocarditis, which may result in fatal disease [12, 14].

Here, we provide the first report to our knowledge of *H. martis* in the Italian peninsula, supported by molecular characterization of parasite isolates and histopathological evaluation of lesions in affected hosts.

## Methods

### Animal and tissue analysis

Carcasses were submitted to the Istituto Zooprofilattico Sperimentale della Lombardia e dell’Emilia-Romagna (IZSLER) in the frame of the wildlife health surveillance plan (Deliberation no. 1763, November 29, 2017) to monitor several parasitic diseases by analyzing vertebrate carcasses. Sixteen *Martes* spp. were examined between 2023 and 2025. One specimen from the Tuscany region was submitted for necropsy and laboratory analyses at the Department of Veterinary Medical Sciences of the University of Bologna in 2024. Examinations included anatomopathological assessment, ectoparasite collection, and tissue sampling.

For this study, ear and spleen samples were collected for subsequent PCR analysis; for freshly deceased individuals, additional tissues were preserved for histopathological assessment, as histological examination is difficult on frozen carcasses. For histopathology, organs from the three recently deceased martens were fixed in 10% buffered formalin, trimmed, embedded in paraffin, sectioned at 3–4 µm, and stained with hematoxylin and eosin (H&E).

### DNA analysis

Molecular analysis was performed on all tissues listed in Table 1. Genomic DNA was extracted using the PureLink® Genomic DNA Mini Kit (Invitrogen, Thermo Fisher), following the manufacturer’s protocol. Screening for apicomplexan blood parasites was performed on 15 subjects using qPCR targeting 18S rDNA, following the protocol of Stanczak et al. [15]. Positive samples were further analyzed by end-point PCR targeting the same locus, as described by Casati et al. [16], which amplifies a ~ 420-bp fragment of apicomplexan *18S* rDNA. Amplicons were sequenced to allow species identification. When *Hepatozoon* sp. were confirmed, an additional end-point PCR was conducted according to Hodžić et al. [12] to amplify a larger (~ 1700-bp) fragment of the 18S rDNA, enabling evaluation of genetic diversity and phylogenetic relationships (see Additional file 1: supplementary Table 1).

**Table 1:** Positive animals and samples analysed in this study

ID sample	Species	Sampling date (dd/mm/yyyy)	Municipality	Region	Tested samples	qPCR	Short-18S PCR and sequencing (% identity)	Long-18S PCR and sequencing	Accession number	Histopathology
Case 1	*Martes foina*	10/06/2024	Anghiari (AR)	T	Spleen	–	–	Yes	PX692507	Spleen
					Lymph node	–	–	Yes		Lymph node^#^
					Heart	–	–	Yes		Heart^#^
					Liver	–	–	Yes		Liver
					Tick	–	–	Yes	PX692508	
Case 2	*Martes foina*	23/05/2025	Fontanelice (BO)	E-R	Spleen	Positive	*Hepatozoon martis* (100% OM256566)	Yes	PX692510	Heart^#^, diaphragm^#^, spleen, liver
Case 3	*Martes foina*	05/09/2025	Pianoro (BO)	E-R	Spleen	–	–	Yes	PX692511	Heart^#^**,** spleen, liver, kidney, lung
Case 4	*Martes foina*	17/04/2023	Sala Baganza (PR)	E-R	Spleen	Positive	*H. martis* (100% OM256566)	No	PX692513	–
Case 5	*Martes foina*	18/04/2023	Quattro castella (RE)	E-R	Ear	Positive	Mixed chromatogram	No	PX692512	–
					Spleen	Positive	*H. martis* (100% OM256566)	No		
Case 6	*Martes foina*	23/05/2023	Valsamoggia (BO)	E-R	Ear	Positive	*H. martis* (99.62% OM256566)	No	PX692514	–
					Spleen	Positive	Mixed chromatogram	No		
Case 7	*Martes foina*	12/10/2024	Monte San Pietro (BO)	E-R	Ear	Negative	–	–	–	–
					Spleen	Positive	*H. martis* (100% OM256566)	Yes	PX692509	
Case 8	*Martes foina*	10/10/2024	Zola Predosa (BO)	E-R	Ear	Positive	Mixed chromatogram	No	–	–
					Spleen	Positive	Mixed chromatogram	No	–	

*E-R* Emilia-Romagna Region; *T* Tuscany Region. *Short-18S PCR* protocol of Casati et al. (2006), amplifying a ~ 420-bp fragment of apicomplexan 18S rDNA. *Long-18S PCR* protocol of Hodžić et al. (2018), amplifying a ~ 1700-bp fragment of the same gene. In the “Histopathology” column, the organs examined are listed for each animal; ^#^organs showing *Hepatozoon* developmental stages; “-" = the corresponding diagnostic technique was not performed

For sample cases 1 and 3, only the latter PCR was performed, as *Hepatozoon* developmental stages had already been detected microscopically in some histological sections. From case 1, an engorged tick was also tested for *H. martis* via the method described in [12]. This tick was first identified morphologically using standard taxonomic keys [17], and molecular identification was then performed by amplifying and sequencing the mitochondrial *COI* gene, following Tuccia et al. [18]. For sequencing, amplicons were excised from the gel, purified using the NucleoSpin Gel and PCR Cleanup kit (Mackerey-Nagel, Düren, Germany), and sequenced on an ABI 3730 DNA analyzer (StarSEQ, Mainz, Germany). Trace files were assembled using Contig Express (VectorNTI Advance 11 software, Invitrogen, Carlsbad, CA, USA), and consensus sequences were compared with published data using BLAST tools (https://blast.ncbi.nlm.nih.gov/Blast.cgi) (accessed on 30 October 2025).

Two datasets were used for running the phylogenetic analysis: one with short sequences of up to 460 bp and a larger fragment of 1660 pb. The former allowed comparison with more *H. martis* sequences available in GenBank®, whereas the latter included more informative sites for phylogenetic analyses. Sequences were aligned in MEGA7 [19] using the MUSCLE algorithm [20] and *Adelina bambarooniae* as the outgroup. Then, the best nucleotide substitution model was calculated in JModelTest 2 [21], and for the short 18S fragment dataset was estimated as Tamura 3-parameter, whereas the longer 18S fragment was Tamura 3-parameter with a gamma distribution of 0.11. Alignments were uploaded into Beauti, where 10^8^ Monte Carlo Markov chains were run with 10% burn-in and a sampling of one every 10^3^ trees. A Bayesian inference phylogenetic tree was built with the BEAST 2.5 package [22], and then Tracer was used to evaluate effective sample sizes and tree convergence. TreeAnnotator estimated the consensus tree; finally, FigTree was used for visualization.

## Results

**Fig. 1:**
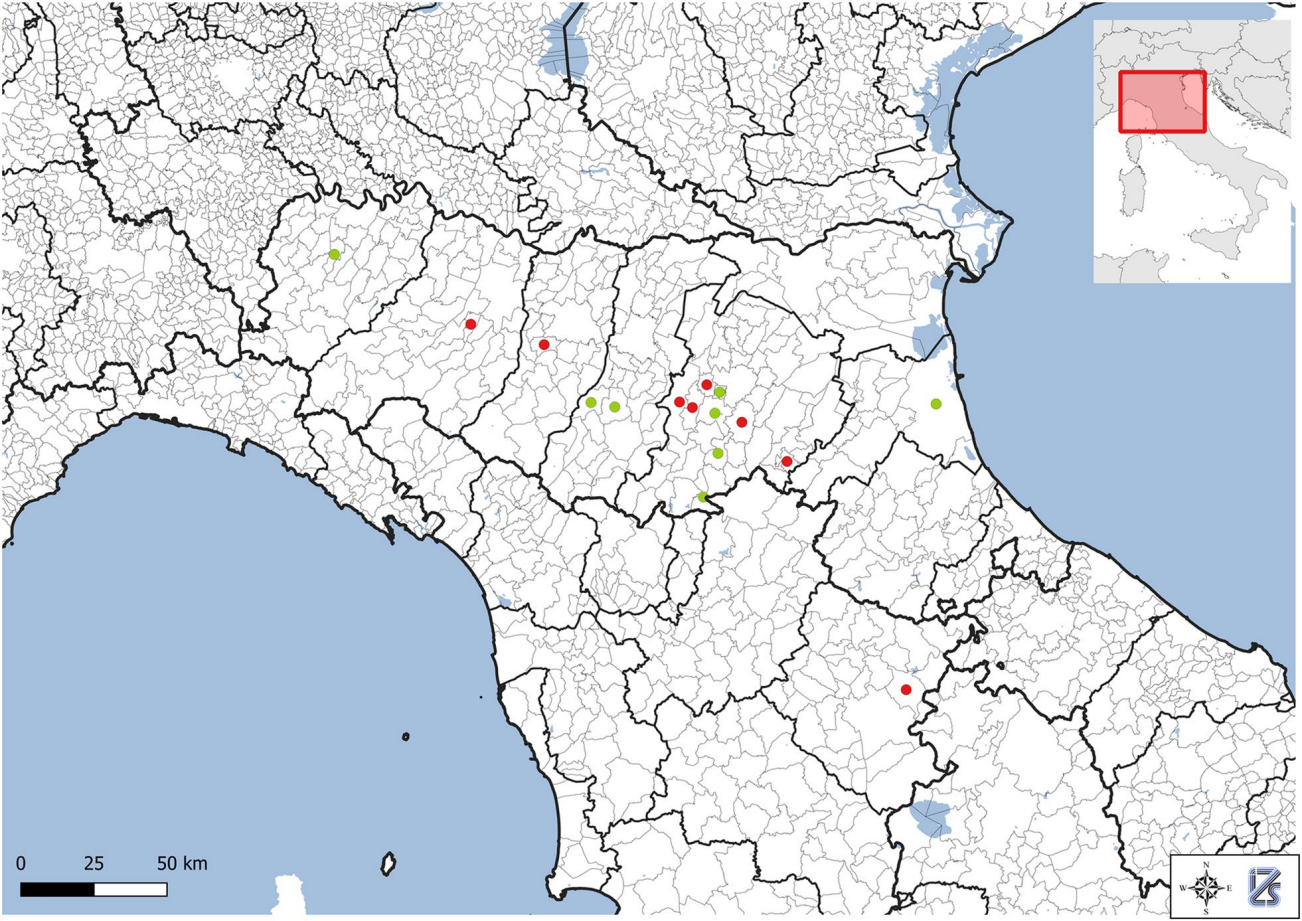
Geographic distribution of tested animals; red dots represent *Hepatozoon martis*-positive samples, while green dots represent negative samples. The figure was generated with QGIS Geographic Information System. Open Source Geospatial Foundation Project. https://qgis.org

Seventeen *Martes* spp. carcasses were examined between 2023 and 2025. Of these, 16 were identified as *M. foina* and one as *M. martes*. Geographically, 16 specimens originated from 6 provinces within the Emilia-Romagna region and 1 from the province of Arezzo (Tuscany), bordering Southeastern Emilia-Romagna (Additional file 1:Fig. 1).

Overall, 8 of 17 (47%) subjects tested positive for at least one diagnostic method, all belonging to *M. foina* species; the only *M. martes* investigated tested negative.

Molecular screening by qPCR detected apicomplexan DNA in 6 of 15 animals. Sequencing of the 420 bp 18S rDNA fragment confirmed the presence of *H. martis* in 5 cases. In three animals, *H. martis* DNA was detected only in spleen samples, whereas in another three, both spleen and ear skin tested positive. However, sequence confirmation was obtained only from the spleen because of mixed chromatograms in the ear tissue. In one spleen sample, sequencing also produced a mixed chromatogram, preventing confident species identification.

In cases 1 and 3, only the long-fragment 18S rDNA PCR [12] was performed after histopathological evidence of *Hepatozoon* sp. developmental stages, yielding positive results from all tested tissues (heart, lymph node, liver, spleen, and tick) and providing complete sequences. Large-fragment sequences were also obtained for case 2 (which also underwent histological analysis) and case 7; BLAST analysis of these sequences showed 99.7–100% identity and 100% query coverage with *H. martis* reference sequences OM256566 (from *Hae. concinna* ticks, Hungary; [8]) and MG136687 (from *M. martes*, Bosnia and Herzegovina; [12]). Long 18S rDNA sequences were deposited in GenBank under accession numbers PX692507-11 and small fragments with accession numbers PX692512-14 (Table 1).

The tick collected from case 1, *M. foina*, was morphologically identified as *Ixodes* sp., since it lacked part of the head, while COI barcoding did not allow exact identification for the unreadable chromatogram.

**Fig. 2:**
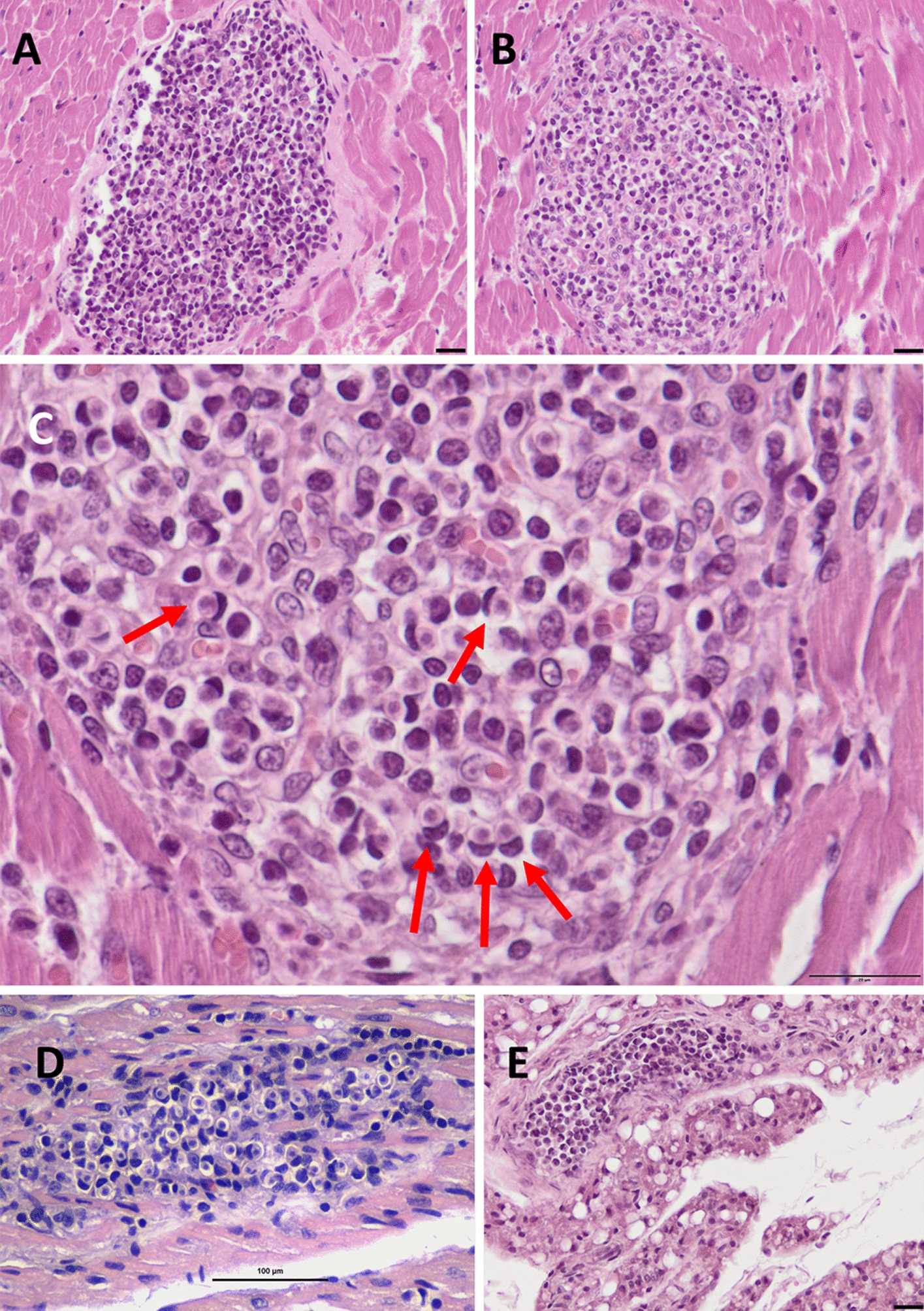
Granulomatous foci containing intracellular zoites observed in various anatomical sites (H&E staining). **A**, **B**, **C**, and **E** case 1; **D** case 2. **A** Granulomatous myocarditis with a well-developed fibrous capsule (scale bar = 20 µm; magnification × 200). **B** Granulomatous lesion with absent or minimal fibrous encapsulation (scale bar = 20 µm; magnification × 200). **C** Higher magnification (× 400) of a granulomatous focus showing intracellular *Hepatozoon* zoites (arrows), which display the typical half-moon-shaped nuclei caused by nuclear displacement following parasite phagocytosis by mononuclear cells (scale bar = 20 µm). **D**, **E** Smaller, less organized granulomatous lesions with numerous parasitized cells in the myocardium (**D**, scale bar = 100 µm; magnification × 400) and perilymph-nodal adipose tissue (**E**, scale bar = 20 µm; magnification × 200). In these lesions, the inflammatory infiltrate (predominantly macrophages) and fibrous response are limited, likely reflecting their more recent development

Histological examination demonstrated the presence of developmental stages of *Hepatozoon* exclusively in the cardiac tissue, the perilymph-nodal brown adipose tissue, and the diaphragmatic muscle tissue. No parasitic stages or associated lesions were observed in the spleen, liver, kidney, or lung. In case 1, the heart showed multifocal granulomatous myocarditis, characterized by well-demarcated inflammatory foci (Additional file 2:Fig. 2 A,B), variably encapsulated (Fig. 2A), consistent with chronicity, or non-encapsulated (Fig. 2B), suggesting a more recent onset. Granulomas were composed mainly of macrophages and lymphocytes, with rare neutrophils. Numerous mononuclear inflammatory cells exhibited crescentic or “half-moon” nuclear displacement due to cytoplasmic engulfment of individual *Hepatozoon* zoites (Fig. 2C), indicative of active phagocytosis.

Analogous granulomatous lesions were observed within the perilymph-nodal brown adipose tissue adjacent to the mandibular lymph node, containing intramacrophage zoites (Fig. 2E) and type II meronts at different stages of maturation (Additional file 3: Fig. 3A).

**Fig. 3:**
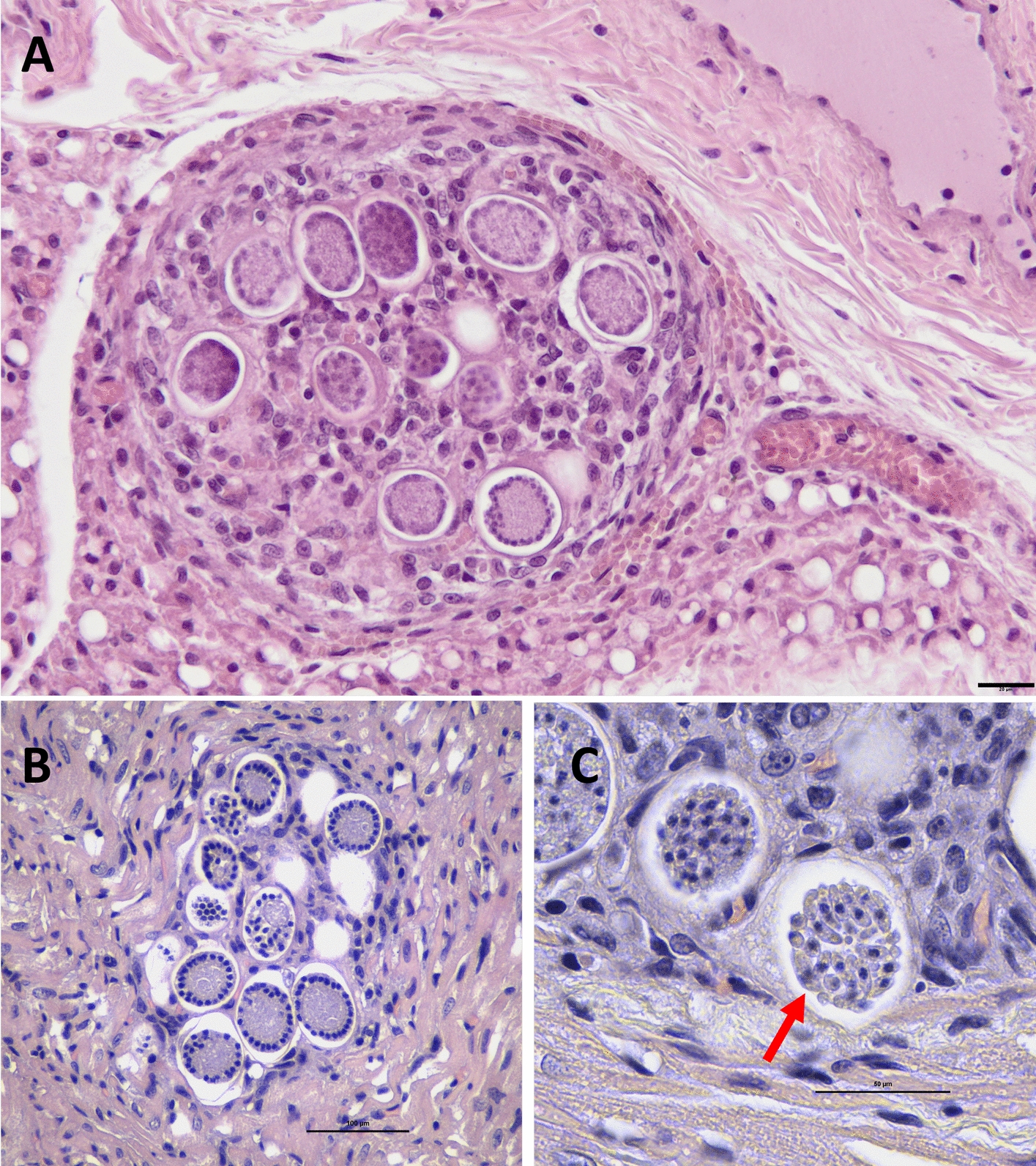
Granulomatous lesions in different anatomical sites showing meronts at various developmental stages. **A** case 1; **B** and **C** case 2. **A** Rounded granuloma in perilymph-nodal brown adipose tissue containing several type II meronts: some with peripheral nuclei and eosinophilic central cytoplasm, others with centrally arranged nuclei (scale bar = 20 µm; magnification × 200). **B** Small myocardial lesion with minimal inflammatory infiltration, showing type II meronts at different maturation stages—less developed forms with peripheral nuclei and more advanced forms with centrally positioned nuclei and nearly mature merozoites (scale bar = 100 µm; magnification × 400). **C** Higher magnification (× 1000, oil immersion) of a myocardial lesion displaying two type II meronts with developed merozoites (arrow) (scale bar = 50 µm)

In case 2, granulomatous myocarditis (Fig. 2D) and diaphragmatic myositis were present, with a more extensive interstitial infiltrate between cardiomyocytes composed mainly of rare neutrophils and plasma cells. Granuloma contained both intracellular zoites and type II meronts exhibiting peripheral or central nuclear arrangements (Fig. 3B), consistent with advanced merogonial maturation. In one lesion, fully developed merozoites were also visible (Fig. 3C). Case 3 showed lesions referable to the previous ones in heart tissue.

Phylogenetic analysis of short 18S rDNA sequences showed that all *H. martis* aligned in the same cluster, and then two separate clusters included *H. felis* sequences with high posterior probabilities. An exception was *H. luiperdjie*, which was placed within the *H. felis* clade (Additional file 4: Fig. 4).

**Fig. 4:**
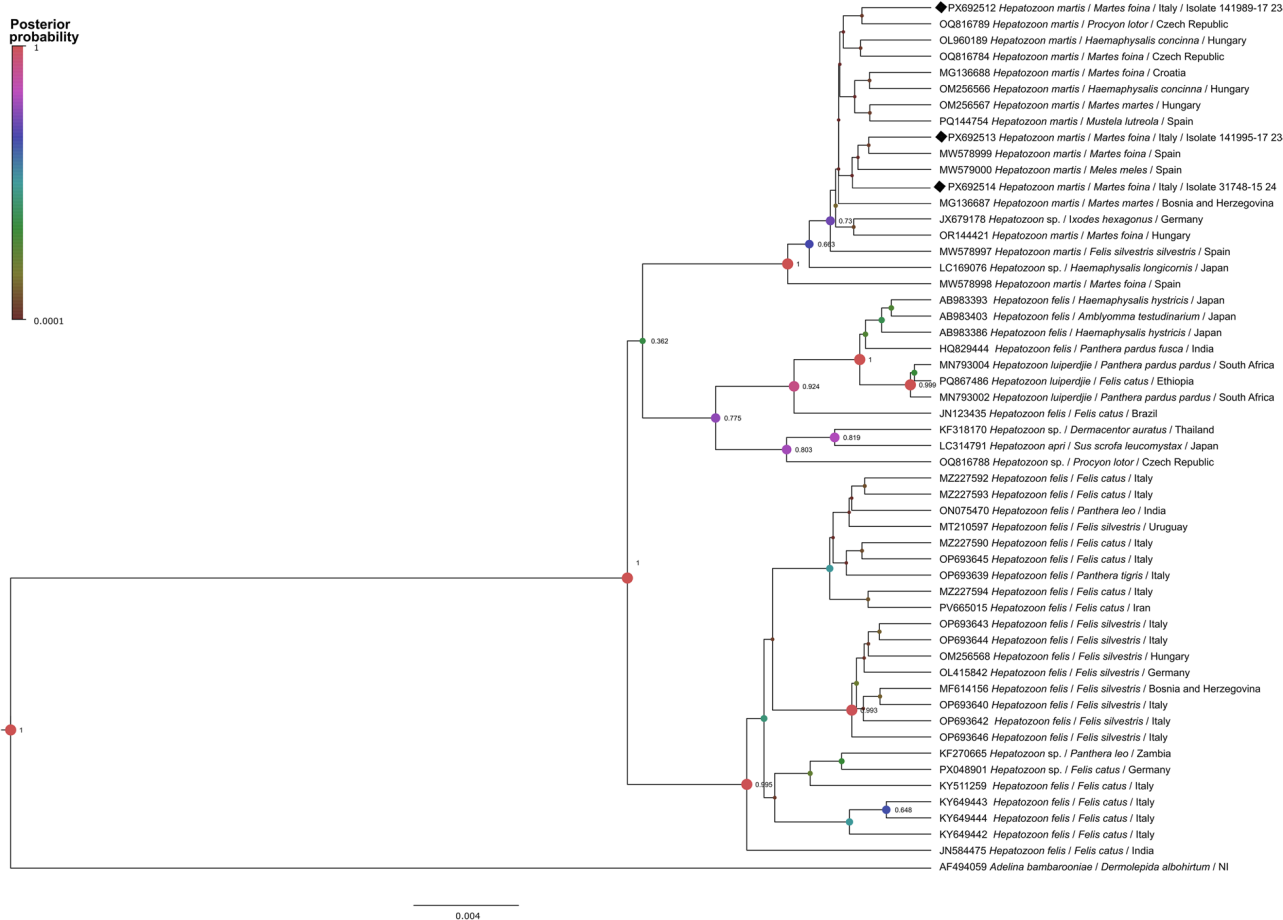
Bayesian inference phylogenetic tree of a 440-bp fragment of the 18S rDNA of *Hepatozoon*. Sequences generated in this study are indicated with black diamonds. Posterior probability values (PPVs) are indicated next to each node, and the size and color of nodes are proportional to these values. PPVs < 0.6 are not shown

Sequences derived from *M. foina* from Italy were grouped with other *H. martis* sequences irrespective of host or geographical origin, i.e. sequences derived from the Czech Republic were mixed with others from Hungary, Spain, or Croatia. In addition, low posterior probability values were observed within the *H. martis* group, suggesting low genetic variability among sequences. The same was observed in the phylogenetic tree constructed for the long 18S rDNA (Additional file 5: Fig. 5), where sequences obtained herein clustered with those of *H. martis* from other geographical regions.

**Fig. 5:**
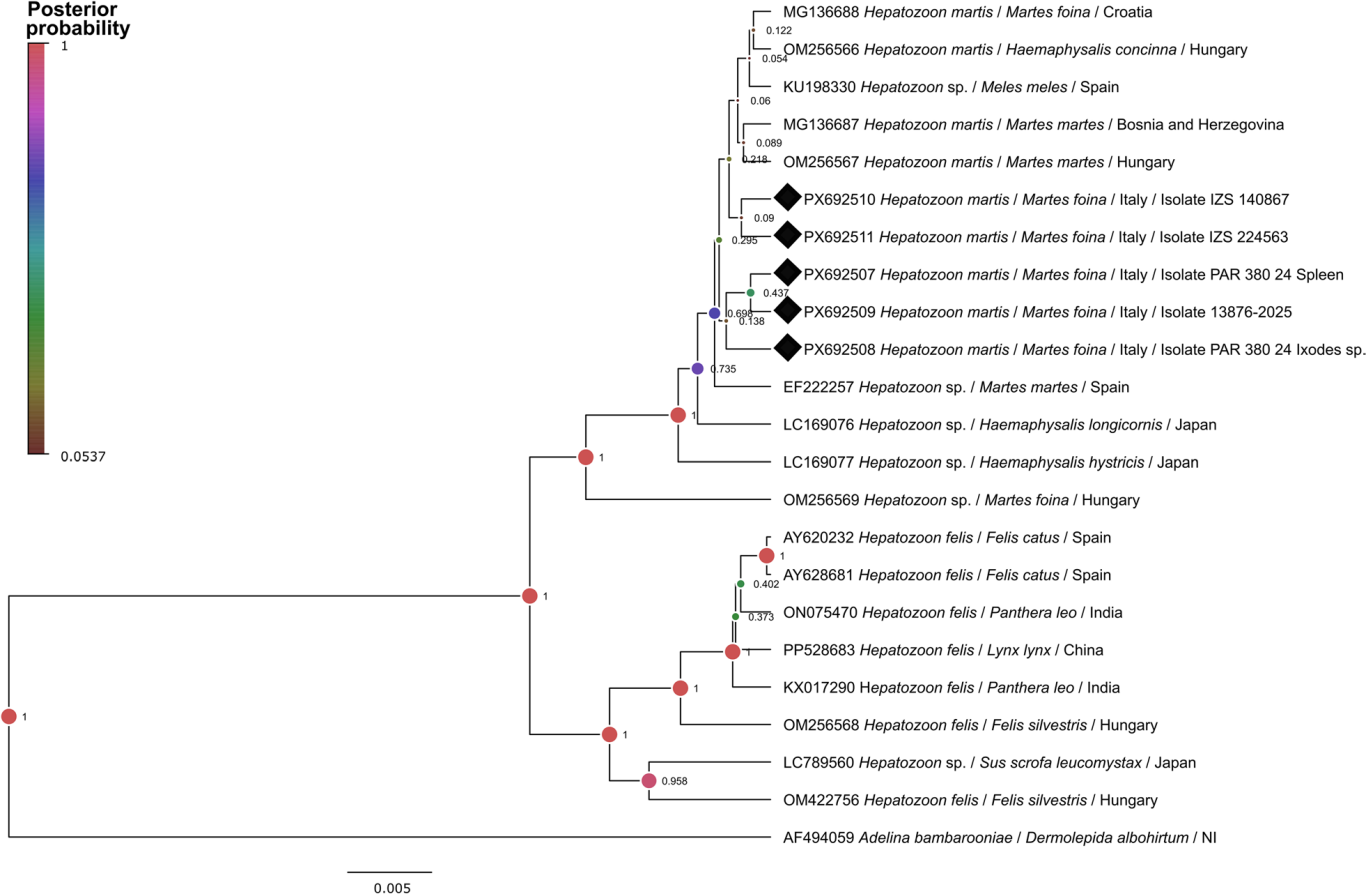
Bayesian inference phylogenetic tree of a 1770-bp fragment of the 18S rDNA of *Hepatozoon*. Sequences generated in this study are indicated with black diamonds. Posterior probability values (PPVs) are indicated next to each node, and size and color of nodes are proportional to these values. PPVs < 0.6 are not shown

A cluster with six sequences from Italy was observed but with a low posterior probability (0.085), whereas the other two generated sequences clustered near a *H. martis* from European pine marten of Bosnia and Herzegovina.

## Discussion

Here, we report the first evidence of *H. martis* infection in Italy in *M. foina*, supported by histopathological analysis of lesions and molecular data and sequencing. In addition, we provide new insights into the merogonic development of *H. martis*, as observed in histological sections of the hearts of naturally infected animals, and describe the histopathological features of the granulomatous lesions caused by the parasite. *Hepatozoon martis* is known to induce a granulomatous inflammation, and granuloma formation in some *Hepatozoon* spp. has been associated with the rupture of meronts and the subsequent host reaction to released merozoites [23].

Previous studies have reported a high prevalence of *H. martis*-related lesions in mustelids across Europe, particularly in European pine martens (*M. martes*) and stone martens (*M. foina*) from Bosnia and Herzegovina, Croatia, and Germany [11, 12, 24].

Our study confirms the observations previously reported by Hodžić et al. [12], namely that *H. martis* shows a predilection for muscular tissue, particularly the myocardium.

Histopathological examination was possible in a limited number of freshly collected animals (3/17), as freezing can induce tissue artifacts that impair the accurate evaluation of microscopic lesions [25].

This may have influenced the estimation of the lesion prevalence relative to molecular positivity, potentially leading to an underestimation of the association between pathogen presence and histopathological changes.

This feature has also been described in other *Hepatozoon* species, such as *H. procyonis* [26], *H. americanum* [27], and *H. felis* and *H. silvestris* [28, 29]. *Hepatozoon* species with a preference for muscular tissue include both cardiac and skeletal muscles, and the associated lesions may have differing pathological consequences for the host. Multifocal granulomatous lesions were frequently observed in the myocardium, skeletal muscles, and occasionally in other tissues such as adipose tissue, tongue, lung, and urogenital organs [12, 24]. In the present study, beyond myocardium, in perimandibular lymph node adipose tissue, multiple granulomatous lesions containing type II meronts or intracellular zoites were seen, expanding the knowledge on the possible anatomical localization of this apicomplexan asexual reproduction.

Similar granulomatous lesions associated with *Hepatozoon* infection have also been documented in martens from Scotland and Korea [14, 30], as well as in minks (*Mustela vison*) from Ontario (Canada) [31] and Pennsylvania (USA), where lesions were sometimes accompanied by concurrent infections such as distemper virus [32]. Based on the myocardial pathological features observed in all histologically examined animals, the high prevalence detected may indicate a condition that compromises the health of infected individuals.

Ortuño et al. [13] detected *H. martis* in 13 stone martens (*M. foina*), one wild cat (*Felis catus silvestris)*, and three badgers (*Meles meles*) from Northern Spain. This infection in badgers had already been reported by Barandika et al. [33] in the same region; however, this record remains in the databases as *Hepatozoon* sp., since those authors were unable to assign the parasite to a definitive species because their study preceded the formal description of *H. martis* by Hodžić et al. [12]. Beyond mustelids, *H. martis* and a closely related *Hepatozoon* sp. have also been identified in the invasive raccoon (*Procyon lotor*) in the Czech Republic [11]. Regarding the invertebrate host, *H. martis* has recently been detected in *Haemaphysalis concinna*, a tick species proposed as a competent vector, in Hungary [8]. Moreover, *H. martis* was the only protozoan detected in ticks collected from mink in Spain, being identified in five *Ixodes hexagonus* pools from La Rioja, including both nymph and female stages [34]. Nevertheless, *Ixodes* spp. are not currently considered competent hosts of *H. martis*. The detection of *Hepatozoon* DNA in a single tick sample in this study is probably attributable to the ingestion of parasitic stages during a blood meal from the infected marten. Indeed, the mere presence of pathogen DNA in a blood-feeding arthropod does not constitute evidence of its competence as a biological vector.

DNA analysis enables the confirmation and characterization of the Hepatozoon spp. involved. In the present study, sequence analysis yielded mixed chromatograms in three cases (Cases 5, 6, and 8), preventing the generation of reliable consensus sequences. This finding could potentially indicate co-infections with different piroplasm species or the presence of distinct genotypes of the same species within individual hosts. However, alternative explanations, such as non-specific amplification or sequencing artifacts, cannot be excluded.

In this sense, the 18S rDNA has been the most employed marker for species identification and has the largest representation in GenBank databases [10]. However, its resolution halts further intraspecific resolution and population structure analyses [35], as observed here with the *H. martis* clade. Sequences obtained from European pine martens from Italy clustered with those from other geographical regions of Europe such as the Czech Republic, Croatia, Hungary, and Spain, suggesting a very low parasite population structure and high gene flow in animal populations, possibly due to high mobility of martens, persistent life cycle stages in definitive hosts, long parasite generation time, or uniform distribution of *H. martis* among hosts [36]. Further sampling of European mustelids, combined with the use of more polymorphic genetic markers, such as mitochondrial and additional nuclear loci, will be essential to clarify these hypotheses and to improve our understanding of the epidemiology and evolutionary relationships of *H. martis*.

## Conclusions

This study reports the first evidence of *Hepatozoon martis* in Italy, confirming *Martes foina* as a natural host through molecular and histopathological analyses. The detection of parasite developmental stages and associated granulomatous lesions, particularly in cardiac skeletal muscle, indicates a marked tissue tropism and suggests potential pathological relevance for infected hosts. Phylogenetic analyses revealed low genetic variability among European *H. martis* isolates, consistent with limited population structure and possible high gene flow. Although ticks have been implicated in the transmission of *H. martis*, vector competence remains unconfirmed, underscoring the need for further studies to clarify transmission pathways and the epidemiological role of wildlife reservoirs.

## Supplementary Information

Supplementary file 1.

## Data Availability

Data supporting the main conclusions of this study are included in the manuscript.
