# Supplementary material for: Hepatozoon martis in Italy: first evidence based on histopathological characterization and molecular confirmation in Martes foina
Source: Parasit Vectors. 2026 Apr 8;19:237. doi: 10.1186/s13071-026-07384-3 (PMC13227661; doi:10.1186/s13071-026-07384-3)
Supplement: Supplementary file 1 [file 13071_2026_7384_MOESM1_ESM.docx]

| molecular method | Target | Sequence (5′–3′) | fragment amplicon length | reference |
| --- | --- | --- | --- | --- |
| qPCR (screening) | 18S rDNA | Bab18S-f: CATGAACGAGGAATGCCTAGT ATG  Bab18S-r: CCGAATAAT TCA CCG GAT CAC TC  Probe BHQ1: -FAM-AAGTCATCAGCTTGTGCAGATTAC GTCCCT- | 116 bp | Stanczak et al. 2015 |
| end point PCR (confirmation) | 18S rDNA | BJ1: GTC TTG TAA TTG GAA TGA TGG;  BN2: TAG TTT ATG GTT AGG ACT ACG. | 420 bp | Casati et al.2006 |
| end point PCR (phylogenetic analysis) | 18S rDNA | HAM-1: GCCAGTAGTCATATGCTTGTC  HPF-2: GACTTCTCCTTCGTCTAAG | 1800 bp | Hodzic et al 2018 |

**Supplementary information**

Additional file 1: Supplementary Table 1: Primers and references of PCR protocols used in this study
